# Micro- and Nanotopography Sensitive Bacterial Attachment Mechanisms: A Review

**DOI:** 10.3389/fmicb.2019.00191

**Published:** 2019-02-21

**Authors:** Yifan Cheng, Guoping Feng, Carmen I. Moraru

**Affiliations:** Department of Food Science, Cornell University, Ithaca, NY, United States

**Keywords:** bacteria-surface interaction, surface sensing, bacteria attachment, nanotopography, microtopography

## Abstract

Bacterial attachment to material surfaces can lead to the development of biofilms that cause severe economic and health problems. The outcome of bacterial attachment is determined by a combination of bacterial sensing of material surfaces by the cell and the physicochemical factors in the near-surface environment. This paper offers a systematic review of the effects of surface topography on a range of antifouling mechanisms, with a focus on how topographical scale, from micro- to nanoscale, may influence bacterial sensing of and attachment to material surfaces. A good understanding of these mechanisms can facilitate the development of antifouling surfaces based on surface topography, with applications in various sectors of human life and activity including healthcare, food, and water treatment.

## Introduction: a Bacterium’s Journey to the Surface

A sessile lifestyle provides bacteria with many advantages, including high nutrient availability and utilization of surface-originated elements in metabolism (Fletcher and Marshall, 1982; Bright and Fletcher, 1983; van Loosdrecht et al., 1990; Tuson and Weibel, 2013). On the other hand, association of bacteria into biofilms lowers their susceptibility to various environmental stresses, including mechanical shear caused by fluid flow, chemical disturbances, or antimicrobial agents (Tuson and Weibel, 2013). While advantageous for the survival of bacterial cells in harsh environments, this resistant form of bacterial life can result in a wide range of adverse consequences for humans, ranging from severe dental and hospital infections (Stoodley et al., 2002), to contamination of food products in processing facilities and subsequent foodborne illness (Scallan et al., 2011). It has been estimated for instance that about 80% of all medical infections are biofilm-derived (Tuson and Weibel, 2013). To effectively fight bacterial biofilms, it is important to understand how bacterial cells attach to material surfaces.

A bacterial cell’s journey to a biotic or abiotic surface is a complex, multistage process that involves locating, approaching, and sensing the proximity of the surface. The transport of bacterial cells to an interface can occur as a result of physical laws such as diffusion (Brownian motion), convective flow or, in the case of motile bacteria, their active movement (van Loosdrecht et al., 1990). Over the course of evolution, the benefits associated with a sessile lifestyle led to the development of surface sensing mechanisms that enable bacteria to detect the presence of solid surfaces using surface-associated chemical gradients, or chemotaxis. These gradients are born from the presence of various chemical species in the aqueous proximity of a surface or the degradation of certain surface components. Such gradients can lead to interactions between bacterial cells and the material surfaces via electrostatic interactions, surface energy, or specific ligand-receptor interactions (Meibom et al., 2004). Metabolic substrates such as amino acids (e.g., aspartate, glutamate, serine, and glycine) (Mesibov and Adler, 1972) and sugar molecules (e.g., glucose, galactose, and fructose) (Adler et al., 1973) are for instance common chemoattractants for *Escherichia coli*. As a bacterium approaches a solid-liquid interface and initiates a reversible attachment to the solid surface, the cell may trap ions and small molecules between its body and the material surface. This process can result in rapid changes in pH (Ponsonnet et al., 2008) and/or osmolality of the confined microenvironment created between the cell membrane and the material surface. Solid surfaces have also been found to influence DNA stability and rate of DNA transformation in bacterial cells (van Loosdrecht et al., 1990). Such changes can potentially be used as cues by bacteria that they are in the proximity of the surface (Tuson and Weibel, 2013), leading up to attachment.

One special case of concentration-based sensing is referred to as “quorum sensing (QS),” in which bacterial cells exchange small extracellular molecular messengers as a means of orchestrating the behavior of a complex microbial community (Ng and Bassler, 2009). Individual cells can sense the status quo of the surrounding microbial community and subsequently adopt strategies for metabolism and survival. For example, quorum sensing was reported to influence attachment of *E. coli* cells to surfaces by altering the cell surface charge (Eboigbodin et al., 2006). Quorum sensing was also reported to be required in the adhesion of *Serratia marcescens*, an opportunistic pathogen and a major cause of ocular infections, to abiotic surfaces (Labbate et al., 2007). Overall, the individual needs of single cells, driven by chemotaxis, and those of the larger bacterial community, broadcasted via quorum sensing, combine and trigger the thrust of bacterial cells toward a surface.

In addition to chemical sensing, bacteria are also capable of picking up surface-associated mechanical cues. For example, bacteria will sense and react to the constrained movement of bacterial appendages in close proximity of a surface or of other cells already attached to that surface (Tuson and Weibel, 2013).

**FIGURE 1:**
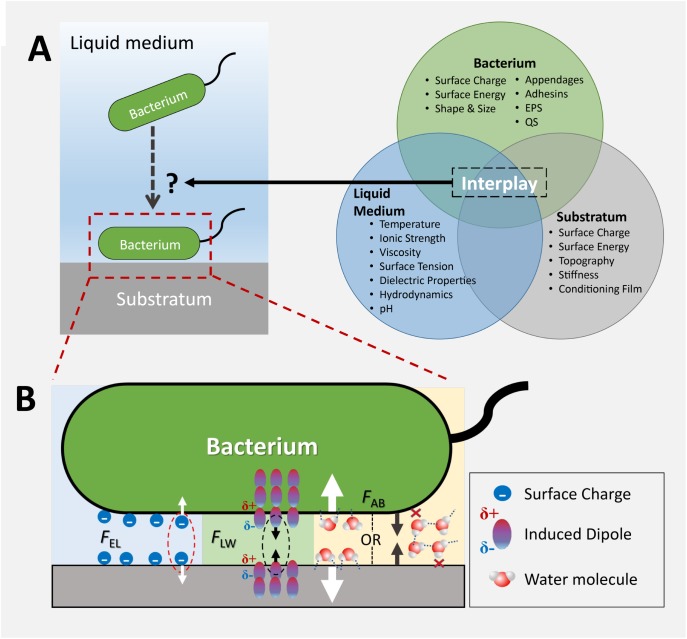
A schematic representation of the factors that influence initial bacterial attachment to a solid-liquid interface. **(A)** The outcome of the attachment process is governed by the interplay between the properties of the bacterium, solid surface, and the liquid medium; **(B)** Physicochemical forces and other factors that affect attachment. EPS, extracellular polymeric substances; QS, quorum sensing; EL, electrostatic interaction; LW, Lifshitz-van der Waals interaction; AB, acid-base interaction. Figure adapted from Cheng and Moraru (2018), with permission.

Although the past decade has witnessed a rapid expansion of knowledge in how bacteria sense a surface, how surface topography of different scale affects bacterial surface sensing and attachment is not yet fully elucidated. This is at least in part due to the fact that bacterial attachment to a surface is a result of the complex interplay between the bacterium, the surface, and the surrounding medium, as illustrated in Figure 1A. Additionally, both the medium-surface interactions and the bacterium-surface interactions are influenced by surface topography. While many studies attempted to interpret the effect of topography on bacterial attachment by singling out one probable anti-attachment mechanism, limited discussion exists regarding the simultaneous involvement of multiple mechanisms in the interaction between bacteria and surface topography. These effects are very important, since textured surfaces are ubiquitous in most natural and man-made environments. The main focus of this paper is to review the current knowledge and understanding of topographical effects in bacteria-surface interactions, with a focus on the importance of topographical scale in these interactions and bacterial attachment mechanisms.

## Roughness: the Good, the Bad, and the Ugly

Surface topography has been shown to significantly impact bacterial attachment and subsequent biofilm formation, and different mechanisms seem to be predominant at micrometric scale vs. nanometric scale (Whitehead et al., 2005; Friedlander et al., 2013; Hsu et al., 2013; Feng et al., 2014, 2015). Before discussing topographical effects on attachment, it is important to first tackle some issues associated with a common descriptor of topography: surface roughness. Roughness is the most deployed parameter for describing surface topography in the biofouling literature (Bollen et al., 1997; Arnold and Bailey, 2000; Medilanski et al., 2002). Often times roughness is used as the sole descriptor of surface topography, largely because of its simple calculation, concise presentation, and prevalence in literature. Surface roughness is represented by different quantitative parameters, including *R*_a_ (the arithmetical average height), *R*_q_ (the root mean square of the height values) and *R*_z_ (the difference in height between the average of five highest peaks and five lowest valleys) (Whitehead and Verran, 2006; Crawford et al., 2012). Therefore, “roughness” of the same surface can vary drastically depending on which roughness parameter is used, as well as the area scanned for roughness characterization (McConnell et al., 2010). Thus, caution should be used when comparing roughness values from different studies.

Studies have shown contradictory results regarding the influence of roughness on bacterial adherence. Some studies found that there was a clear relationship between roughness and bacterial adherence (Bollen et al., 1997; Arnold and Bailey, 2000; Medilanski et al., 2002) while others did not find such correlations (Vanhaecke et al., 1990; Boulangé-Petermann et al., 1997; Tide et al., 1999; Flint et al., 2000; Verran and Boyd, 2001; Table 1). Many factors could have contributed to such contradictory results, including experimental variance of roughness measurements, physiological differences between the bacterial species tested, or the fact that other surface physicochemical properties that could have influenced bacterial behavior were not considered. As it will be detailed in a later section, roughness can affect various physicochemical properties of a surface, such as surface wettability. The dependency of surface wettability on the interaction between roughness and hydrophobicity could potentially lead to conflicting conclusions regarding the effect of roughness on bacterial attachment.

**Table 1:** Influence of surface roughness on bacterial attachment.

Surface material	Roughness	Influence on attachment	Microorganisms	Topography defined?	Reference
Stainless steel with different finishes	9–145 nm (*R*_a_)	Higher attachment on rougher surfaces	Indigenous bacteria from poultry rinse	No	Arnold and Bailey, 2000
Stainless steel	0.03–0.89 μm (*R*_a_)	Attachment increased with higher roughness; bacteria tend to align with scratches of similar dimension	*P. aeruginosa, P. putida, D. desulfuricans, Rhodococcus* spp.	Partially	Medilanski et al., 2002
Titanium implant	0.81 and 0.35 μm (*R*_a_)	Rougher surfaces harbored 25 times more bacteria	Indigenous oral microbiota	No	Bollen et al., 1997
Stainless steel	0.01–1 μm (*R*_a_)	No statistical difference	*S. thermophilus*	No	Boulangé-Petermann et al., 1997
Stainless steel	0.5–3.3 μm (*R*_a_)	No difference	*S. thermophilus* and *S. waiu*	No	Flint et al., 2000
Stainless steel	0.66–1.2 μm (*R*_a_)	No difference	*L. monocytogenes*	No	Tide et al., 1999
Stainless steel	0.1–0.9 μm (*R*_a_)	Smoothest surface had 100 times lower attachment than the roughest surface, but the difference was minimal for hydrophobic strains	*P. aeruginosa*	No	Vanhaecke et al., 1990

It is also important to point out that surfaces with drastically different topography can have similar roughness parameters. Roughness describes only the height variation of a surface, whereas topography represents the configuration of a surface in a three-dimensional space, often characterized by vertical features (e.g., protrusions and recessions) and their spatial arrangement. Being an amplitude parameter, roughness does not capture any spatial information including the geometric details, density, periodicity, symmetry or hierarchical arrangement of surface details, many of which have been shown to play a critical role in bacterial attachment (Crawford et al., 2012). In addition to roughness, spatial parameters (e.g., summit density and power spectral density) and hybrid parameters – which consider aspects of both amplitude and spatial characteristics – can be used to provide a more comprehensive characterization of surface topography (Stout et al., 2000; Crawford et al., 2012). Another factor that could confound the influence of roughness on attachment is the lack of differentiation between bacterial adhesion to a surface and retention by the surface. Retention is a description of both initially attached and the cells that remain undetached, despite external stresses (i.e., shear caused by flow), while adhesion refers to the former. Therefore, it is not always clear if bacterial cells present on a surface is the result of attachment or ineffective detachment due to the protection provided by rough surfaces (Scheuerman et al., 1998). Roughness may sometimes just be a minor factor for adhesion, but facilitates retention (Bos et al., 1999).

Because of these limitations of roughness, an increasing number of studies are being conducted on surfaces with precisely defined topography, in an attempt to uncover the general principles underlying the effect of surface topography on bacterial attachment. Some of these findings are summarized in Table 2. However, no general trend is observed when analyzing these data either. Possible causes for the mixed results may include, among others: (1) a change in surface topography is often accompanied by changes in physicochemical properties of the surface; and (2) differences in the size and shape of the various elements of surface topography compared to those of the bacterial cells. In the next section, the effect of topographical scale, from micro- to nanoscale, on bacteria-surface interactions and subsequent cell attachment will be reviewed.

**Table 2:** Bacterial attachment behavior on surfaces with defined or partly defined topography.

Surface material	Topography	Influence on attachment	Microorganisms	Reference
Stainless steel	Attachment inducing surfaces had 0.7 μm trenches	Higher attachment; cells tend to align with trenches	*P. aeruginosa, P. putida, D. desulfuricans, Rhodococcus* spp.	Medilanski et al., 2002
Polydimethylsiloxane (PDMS)	Post-array with diameter of 300 nm to 1 μm, and interstitial distance of 0.8–4 μm	Attachment depends on spacing between posts, which was close to the dimensions of bacterial cells	*P. aeruginosa, E. coli*, and *B. subtilis*	Hochbaum and Aizenberg, 2010; Epstein et al., 2011
Silica, alumina	Silica wells of 0.5 μm dia wells and 0.2 μm interwell spacing; 1 × 1.5 μm rectangles with interwell spacing of 2 μm; 1 × 2 μm rectangles with interwell spacing of 0.5 μm; depth of all wells 27–32 nm; alumina with 20 or 200 nm dia pores	Bacterial cells tend to bind to features in a way that maximizes contact area	*E. coli*, *L. innocua* and *P. fluorescens*	Hsu et al., 2013
PDMS	Hexagonal features of 2.7 μm in height, 3 μm in diameter, separated by 440 nm trenches	Adhesion to topographic surfaces was reduced compared with flat controls; flagella appeared to help explore trenches where bacterial cells did not have access, facilitating attachment	*E. coli*	Friedlander et al., 2013
Silicon	Rectangular grooves of 10, 20, 30, and 40 μm in width and 10 μm in depth; testing under flow conditions	Attachment independent of groove width; motile strains could reach and accumulate on the bottom of grooves, while the nonmotile strain could not.	*P. aeruginosa*, motile and nonmotile *P. fluorescens*	Scheuerman et al., 1998

## Effect of Topographical Scale on Bacterial Attachment

**FIGURE 2:**
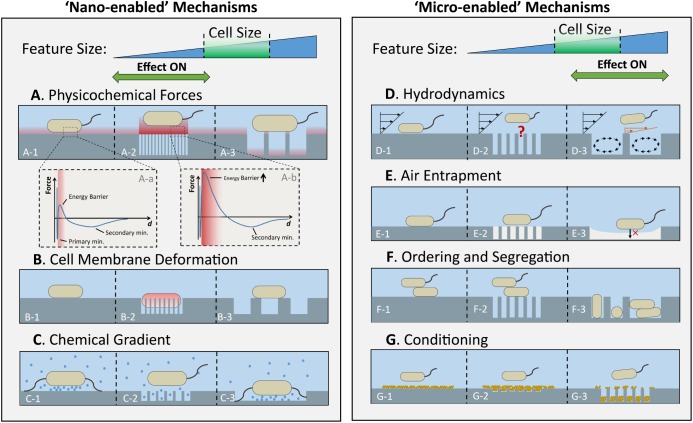
Scale-dependent effects of surface topography on various factors that influence initial bacterial attachment. The anti-attachment effects of physicochemical forces **(A)**, cell membrane deformation **(B)**, and chemical gradient **(C)**, are enhanced by surfaces with features much smaller than bacterial cells (left panel), whereas the anti-attachment effects of hydrodynamics **(D)**, air entrapment by topographic features **(E)**, and cell ordering and segregation **(F)** are enhanced by the topographies with feature sizes larger than or comparable to bacterial cells (right panel). Within each row, from left to right, the scenarios for a flat surface, a surface with nanoscale, and a surface with microscale topography are illustrated. The typical force-distance profile for a flat and a nanoporous surface, respectively, are illustrated in A-a and A-b. Conditioning films **(G)** complicate attachment trends by altering the chemistry and topography of the neat surface.

Previous reviews on the effect of micro- and nanoscale topography on bacterial attachment provide a good summary of the most relevant work in this field (Anselme et al., 2010; Graham and Cady, 2014; Meng et al., 2014; Hasan and Chatterjee, 2015; Serrano et al., 2015). Several studies have shown that bacterial attachment can be controlled to a certain degree using patterned surfaces featuring repeating topographical elements of sizes ranging from nanometers to micrometers. Yet, understanding of the underlying mechanisms of attachment is still limited. Here, we intend to provide a mechanistic analysis of the effects of surface topography on bacterial sensing and attachment. The main mechanisms and their possible effects, concerning topographical scale, on attachment are represented schematically in Figure 2, as follows: physicochemical forces (Figure 2A), cell membrane deformation (Figure 2B), chemical gradients at the solid-liquid interface (Figure 2C), hydrodynamics (Figure 2D), surface wettability and air entrapment (Figure 2E), topography-induced cell ordering and segregation (Figure 2F), and conditioning film (Figure 2G).

### Influence of Surface Topography on Physicochemical Forces

Bacterial attachment can be explained to some extent by physicochemical bacteria-surface interactions. The thermodynamic theory, the classical DLVO (Derjaguin-Landau-Verwey-Overbeek) theory, and the extended DLVO (or XDLVO) theory are the most commonly used physicochemical approaches used to describe bacteria-surface interactions. However, the biological, chemical and structural complexity of bacterial cells, superimposed onto sometimes, similarly, complex substrate properties, pose great challenges toward the development of a unified physicochemical theory of bacteria attachment. In a comprehensive review of this topic, Bos et al. (1999) provided a comparison of the three theories mentioned above and their respective limitations.

In the classical DLVO theory, the total interaction force between a surface and a bacterium cell is given by the sum of Lifshitz-van der Waals (LW) attractive forces and electrostatic (EL) interactions (Marshall et al., 1971). The Van der Waals forces are dominant in the vicinity of the surface, but decrease sharply with separation distance *h* as *h*^−3^, while the Coulomb electrostatic interactions become dominant further away from the surface (Hori and Matsumoto, 2010). Bacteria cells and natural surfaces in aqueous solutions are usually negatively charged. This gives rise to a repulsive electrostatic energy that increases as the ionic strength of the surrounding aqueous medium decreases (van Loosdrecht et al., 1989). At low ionic strength, when a bacterial cell approaches a surface, it encounters an energy barrier that cannot be overcome solely by motility or Brownian motion; at high ionic strength, this energy barrier vanishes and bacterial cells can easily approach the surface and adhere irreversibly (Bunt et al., 1995; Vigeant and Ford, 1997; Otto et al., 1999; Bolster et al., 2001; Hori and Matsumoto, 2010).

The DLVO theory was later modified by van Oss into the extended DLVO (XDLVO) version (van Oss, 1993). The XDLVO theory accounts for both interactions described above, as well as for short range interactions, including hydrogen bonding an ion pair formation, are summarized as acid-base (AB) interaction forces (van Oss, 1993) (Figure 1B). The acid-base interaction forces are dominant in the short-range, at separation distances of less than 1 nm between bacteria and the surface, but they diminish exponentially in magnitude as the separation distance increases (van Oss, 1993). The total interaction force between the surface and the bacterium, $F_{\text{Total}}^{\text{XDLVO}}$, can be calculated as the vector sum of the Lifshitz-Van der Waals attraction (F_LW_), the acid-base interaction (F_AB_) and the electric double layer interactions (F_EL_) (Li and Logan, 2004):

(1) $$F_{\text{Total}}^{\text{XDLVO}}=F_{\text{LW}}+F_{\text{EL}}+F_{\text{AB}}$$

In general, the LW interactions are attractive, whereas the EL interactions can be attractive or repulsive, depending on the sign of electrical charges on the surface and bacteria, respectively. The AB interactions are a result of electron acceptor/electron donor interactions between polar moieties. Depending on the polarity, or the hydrophobic-hydrophilic properties, these interactions can be attractive (hydrophobic attraction) or repulsive (hydrophilic repulsion or hydration effects) (Figure 1B), and their magnitude may be up to 100-fold greater than that of EL and LW (Bos et al., 2000; Feng et al., 2014). The XDLVO theory was found to be more accurate in predicting initial attachment of colloidal particles (Brant and Childress, 2002a,b; Brant, 2004) and bacteria (Bayoudh et al., 2009) than the DLVO theory, due to the incorporation the acid-base interactions, which can significantly affect the direction of total force within a few nm separation distance between the bacteria and a surface.

The total bacterium-surface interaction force calculated using Equation (1) can be plotted as a function of separation distance, resulting in curves similar to the ones in Figure 2A. A typical force-distance curve for bacterium-surface interactions has two important features: the peak representing the energy barrier, and the secondary minimum (Figure 2A-a,b). Energy barriers have been found to impede bacterial attachment of a wide variety of bacteria by “blocking” the cells from approaching the surfaces (Morisaki et al., 1999; Li and Logan, 2004; Feng et al., 2015). The secondary minimum, on the other hand, reflects a restriction of the bacterial movement by “trapping” the cells in an energy well (Van Der Westen et al., 2018). Bacterial cells are not able to overcome the energy barrier solely by their motility or Brownian motion, yet their appendages have been hypothesized to penetrate the energy barrier due to their small radii, and then effectively bridge the cell and substratum (Hori and Matsumoto, 2010). This phenomenon has been confirmed experimentally using total internal reflection microscopy (Van Der Westen et al., 2018).

It should be noted that the original DLVO or XDLVO modeling of bacterial attachment was developed based on ideally smooth surfaces (Bhattacharjee et al., 1996), and hence it is not directly applicable to surfaces with topography. Next, we will showcase some approaches for adapting these physicochemical theories to account for surface topography.

The surface element integration (SEI) technique (numerical) and Derjaguin’s integration (analytical) are two common methods used to incorporate the effect of topography in particle-substratum interactions (Bhattacharjee and Elimelech, 1997; Hoek et al., 2003; Hoek and Agarwal, 2006; Martines et al., 2008). Hoek and Agarwal (2006) simulated colloid-surface interactions using both SEI and the Derjaguin’s integration method. They found that the properly weighted average of the analytical expression of Derjaguin reasonably approximates the predictions by the SEI model. The magnitude of the interaction energy was lower on textured surfaces compared to smooth counterparts with identical chemical properties (Hoek and Agarwal, 2006). It was shown that nanoscale hemispherical asperities with diameters between 10 and 90 nm and an aspect ratio ∼1.0 on a repulsive surface may create locally attractive sites for particle deposition (Hoek and Agarwal, 2006). These sites often coincide with the valleys where LW-induced attractive energy wells are present (Hoek et al., 2003). The suggested mechanism for these findings is that the surface protrusions physically bar the particles from accessing the majority of the substrate surface, consequently increasing the mean separation distance and therefore weakening the repulsive energy barrier (Hoek et al., 2003). Using a similar methodology, Martines et al. found that asperity diameter was the most influential parameter on total interaction energy for surfaces with nanometric pits, cylindrical and hemispherical pillars of diameters of 20–200 nm and depth of 20–150 nm, with an aspect ratio ∼1.0 (Martines et al., 2008).

A joint research team from Cornell University and Rensselaer Polytechnic Institute has used anodization to manufacture surfaces with nanoscale features of controlled geometry and size, and high aspect ratio (up to about 150) (Feng et al., 2014, 2015). Nanoporous anodic aluminum oxide (AAO) surfaces with pore sizes of 15 and 25 nm significantly reduced attachment by *E. coli*, *Listeria* spp., *Staphylococcus aureus*, and *Staphylococcus epidermidis* compared to a nanosmooth control, while surfaces with pore diameters of 50 nm or larger increased attachment compared to both the smaller pore size surfaces and the nanosmooth control. The authors calculated total interaction forces by integration over the surface area of the cylindrical pores (Feng et al., 2014). As illustrated in Figure 2A, the large surface area originating from the vertical surfaces of the densely-packed, small diameter pores of the 15 and 25 nm pores greatly enhanced the magnitude of *F*_EL_ and *F*_AB_ (Figure 2A-2), and consequently the total energy barrier a bacterium had to overcome (Figure 2A-b) was much larger compared to a flat surface (Figure 2A-1,A-a). Surfaces with larger pores, on the other hand, imposed lower energy barriers against bacteria, because their smaller external surface area resulted in much weaker *F*_EL_ and *F*_AB_ (Figure 2F-3) compared to the smaller pore surfaces, or even that of a flat surface. In a follow-up study, this group demonstrated how this predictive approach can be used to optimize the different elements of surface topography, particularly pore diameter and density, to further enhance the bacteria repelling effects of nanoporous surfaces (Feng et al., 2015).

### Interplay Between Bacteria Related Factors and Surface Topography

Surface topography can affect the expression of bacterial adhesins. For example, it was reported that *E. coli* cells that adhered to nanostructured gold surfaces (roughness *∼*100 nm) underwent suppression of type-I fimbrae synthesis and upregulation of stress response genes compared to those that adhered to flat counterparts (Rizzello et al., 2012, 2013). Rizzello et al. (2012) found that nanoroughness triggered over-expression of *cpxP* and *degP* of the Cpx two-component system, which is activated by the presence of large amounts of misfolded fimbrial protein aggregates associated with the inner membrane. Such findings clearly demonstrate that bacteria are able to actively sense and respond to surface topography, which in turn affects attachment.

More recently, a mechanical bactericidal mechanism has been associated with nanopillars that can kill bacteria by rupturing or deforming bacterial cell membranes, resulting in flattened cell morphology as observed by scanning electron microscopy (SEM) or atomic force microscopy (AFM) (Ivanova et al., 2012, 2013; Dickson et al., 2015; Hasan et al., 2015). This bactericidal mechanism is analogous to having bacteria lying on a “bed of nails” (Figure 2B-2). Some of these nanopillar topographies were inspired by nature, for instance the surface of cicada wings, which are covered with nanopillars that can kill *Pseudomonas aeruginosa* within minutes of contact (Ivanova et al., 2012). Bacteria killing by natural topography was also reported for dragonfly wings (Ivanova et al., 2013). An alternative “ripping” model has been proposed to explain the killing of *E. coli* by the uneven nanopillars of dragonfly wings, where bacterial membranes are “ripped” by the shear forces caused by the movement of the cells affixed to the nanopillars (Bandara et al., 2017). In contrast, when a bacterium lands on a surface with microscale topography, the larger contact area between the cell and the material surface results in a contact pressure too low to cause significant membrane deformation (Figure 2B-3).

Efforts have been made to translate these findings and the wisdom of nature’s design into man-made biomimetic surfaces (Dickson et al., 2015; Hasan et al., 2015). Dickson et al. (2015) successfully imprinted nanopillared topography of cicada wings onto poly(methyl methacrylate) (PMMA) films. After exposing *E. coli* cells to nanopillared PMMA (feature width 60–215 nm, spacing 100–380 nm), the authors found that pillared surfaces both reduced the density of adherent cells compared to a flat control, and killed a greater fraction of the cells that did adhere, with 16–141% higher death rate than on the control surfaces. Furthermore, nanopillars with smaller diameter and narrower spacing performed better at killing bacteria. This suggests that specific nanoscale topographies represent a promising route for engineering bactericidal surfaces without the use of toxic compounds.

Notably, bacteria can employ a variety of mechanisms to colonize terrains with diverse surface topographies. Certain species (e.g., *S. epidermidis*) produce surface associated adhesins that facilitate cell-cell cohesion, allowing early colonizers to provide a foothold for later arrivals. Cell-cell interactions can also occur between bacteria from different species, leading to the development of symbiotic microbial biofilm communities. For instance, in the process of dental biofilm formation, *S. gordonii* is one of the primary colonizers to a conditioned tooth surface, followed by other bacterial species (Rickard et al., 2003). These “pioneers” provide more favorable adhesion conditions for the subsequent colonizers compared to the actual surface.

Bacterial appendages were consistently shown to interact directly with surface topographys. Friedlander et al. (2013) reported that flagellated cells explored recessed topography inaccessible to cell bodies using their flagellar filaments; furthermore, such filaments were able to form networks that bridged the large inter-feature spacings like a “hammock,” resulting in improved attachment of additional cells. Appendages can also help microorganisms recognize structures protruding from substrates. For instance, single cells of *Shewanella oneidensis* could recognize patterned silicon nanowires of 300 nm in diameter and 3–15 μm in height; propelled by appendages, the cells were able to move toward the nanowires and initiate attachment (Jeong et al., 2013).

The appendage-mediated surface adaptation by bacterial cells also plays a role in counteracting some effects of topography-induced cell ordering and segregation, which will be discussed in section Topography-Induced Cell Ordering, Segregation, and Removal.

### Conditioning Films and Chemical Gradients

Solid surfaces immersed in a liquid are typically covered by an adsorbed layer consisting of various molecules, termed a “conditioning film.” The adsorption of macromolecules (i.e., proteins) to the surface is governed by the interplay of various physicochemical factors, which will be discussed in the following section. Efforts to understand the effects of this conditioning film on bacterial attachment have led to contradictory findings. Some studies reported an inhibitory effect of the conditioning film on attachment by *Pseudomonas* spp., *L. monocytogenes*, *E. coli*, *S. aureus*, *S. marcescens*, vegetative cells and spores of *Bacillus* spp. (Fletcher, 1976; Pringle, 1986; Barnes et al., 1999; Parkar et al., 2001; Garrido et al., 2013), while others found the opposite (Jullien et al., 2008; Koo et al., 2010; Hwang et al., 2013). These mixed results could be due to the wide range of bacterial strains used, as well as the different testing conditions. The macromolecules adhered to a surface can significantly modify its physicochemical and topographical properties, thus leading to unpredictable deviations from the anticipated outcome of bacteria-surface interactions. Some mechanisms by which conditioning films can affect attachment include:

(1) *Masking/changing surface properties of the neat material surface*. Bakker et al. (2004) exposed polyurethane coatings of different roughness, hydrophobicity, and elasticity to natural seawater, for 1 h, to study the effect of the conditioning film on bacterial attachment. They found that after this exposure the water contact angles on hydrophobic and hydrophilic polyurethane converged to an intermediate level; this was accompanied by elevated surface nitrogen concentration, indicating the adsorption of proteins onto these surfaces. Upon adsorption, proteins may undergo conformational changes at the liquid-solid interface, to lower the total free energy of the system; the extent of unfolding is dependent on the hydrophobicity of the solid surface, which makes the surface energy after conditioning less predictable (Wu et al., 1986; Jones and Fernandez, 2003). Long exposure times may lead to complete masking of the underlying surface. For example, the composition of the conditioning film was reported to become independent of surface properties after only 4 h of exposure (Maki et al., 1990).

(2) *Modification of surface topography*. Both surface-smoothening and surface-roughening effects have been reported as a consequence of surface conditioning. Bakker et al. (2004) reported that the mean surface roughness of polyurethane surfaces increased on average by 4 nm after 1 h exposure to natural seawater; on the other hand, surface roughness of orthodontic composite resins was significantly reduced in the presence of salivary conditioning films (Mei et al., 2011). These different effects may be due to the different substrata and liquid media used in these studies. Aggregation of proteins at the liquid-solid interface may also occur when cohesion between the adherent protein and incoming protein is thermodynamically more favorable than adhesion to the bare substratum (Perevozchikova et al., 2015), which can cause surface roughening. Surface topographies with nanometric height and/or depth are particularly susceptible to complete masking of the topography by conditioning films (Figure 2G-2), as the dimensions of adsorbed macromolecules (i.e., proteins) are also in the nanometer range (Anselme et al., 2010). Surface topographies with feature sizes in the micrometric range are more resistant to such masking effects (Figure 2G-3).

(3) *Providing sites for specific bacteria-surface interactions*. The protein coating of abiotic surfaces can also provide sites to which bacteria may bind via highly specific receptor-ligand interactions (Hermansson, 1999). Adhesion forces measured using atomic force microscopy (AFM) in the presence of specific bonds between bacteria and a surface covered in biomolecules were 2–3 times stronger than in the absence of such contributions (Busscher et al., 2008).

These mechanisms are further complicated in the presence of certain nanoscale surface topography that can alter the kinetics of protein adsorption. Lazzara et al. (2011) demonstrated that highly porous AAO substrates (pore diameters of 20–80 nm and 0.8–9.6 μm pore depth) act as a highly efficient sink for proteins in the surrounding liquid phase, resulting in a much slower protein coverage rate and therefore a longer time to achieve complete surface coverage compared to a flat, nonporous surface. These changes in surface coverage by proteins (Figure 2G), as well as the protein concentration profile at the solid-liquid interface (Figure 2C) can significantly affect how bacteria sense the solid substrate. For surfaces with nanoscale topography, the high specific surface area (surface area per unit volume) can significantly alter adsorption kinetics of biomolecules to the surface, by greatly increasing the number of sites accessible for adsorption (Xu et al., 2014). When high aspect ratio nanoscale pores are present (Figure 2C-2), the chemical species that would otherwise be confined to a thin liquid film between a bacterial cell and the substrate can diffuse through the nanopores (Vázquez et al., 2015). Consequently, the concentration-sensitive signal transduction pathways might not be triggered to the same extent as in the case on a flat surface (Tuson and Weibel, 2013). Thus, the nanoporous topography might conceal some cues commonly utilized by bacteria for surface sensing, such as proton (pH) gradient and osmolality (Tuson and Weibel, 2013; Figure 2C-1). If the scale of the topographical details is comparable to that of the cell, confinement of the cell within the structural elements of the microscale topography may take place, but the concentration-sensitive surface sensing mechanisms that initiate bacterial attachment might not be disturbed (Figure 2C-3).

### Impact of Hydrodynamics

More often than not, bacteria in natural or man-made environments are subjected to flow conditions, and thus hydrodynamics plays a significant role in their initial attachment. While under static fluid condition the impact of the surrounding liquid is limited to hydrostatic pressure, under flow conditions bacteria experience hydrodynamic forces that influence their motion (translational and angular velocity) or deformation (extensional strain and shear strain). Thus, the hydrodynamic environment must be considered in any bacterial attachment study conducted under flow conditions. It is also important that the actual surface topography is considered in such studies, because local flow rate and strain-stress distribution are sensitive to the shape and distribution of the elements of surface topography (Halder et al., 2013).

Surface topography at the microscale has been found to considerably modify the near-surface microfluidic environment, which influences the hydrodynamic force fields experienced by cells during their initial settlement (Halder et al., 2013, 2014; Lee et al., 2013). Using computational fluid dynamics (CFD), Lee et al. (2013) simulated the crossflow patterns through a filtration membrane with parallel prism-like topography (spacing = 400 μm, height = 200 μm) in the laminar flow regime, and reported higher local shear stress near the apex of the ridges than in the troughs where vortices formed (Figure 2D-3). This simulation result corroborates with their experimental observation that lower deposition of bacteria occurred in the upper region of the prism-like topography than in the troughs, suggesting that micro-topography alters bacterial attachment patterns by triggering variation in local flow. Similarly, Halder et al. (2013) investigated the near-surface microfluidic environment developed on a surface patterned with microwell arrays (diameter 1–10 μm, spacing of 2 and 5 μm), and their influence on initial settlement of *E. coli* cells. CFD simulations revealed that structural features with narrower spacing were associated with sharply fluctuating stress-strain rate along the periphery of the microwells. A follow-up study conducted by the same group tracked the dynamics of *E. coli* cells over such microwell-patterned surfaces, and demonstrated an increased velocity of cells compared to that of cells over a flat surface (Halder et al., 2014).

Halder et al. (2013) have shown that surface coverage by *E. coli* under flow conditions increased from 2% on 10 μm microwell-patterned surfaces to about 25% on 1 μm microwell-patterned surfaces, similar to the trends observed under static conditions. Hence, the differences in coverage between the two surfaces could not be entirely attributed to topography-induced differences in hydrodynamic conditions, and other factors whose effects are correlated with topographical scale, for instance physicochemical forces (Figure 2D), might be responsible for this effect. Nonetheless, it is possible that the impact of topography on the shear stress or shear rate near the surface may diminish with the size of surface topographical elements, as some minimal space is probably necessary for certain microfluidic patterns to develop. As of now, no mechanistic study on the effect of nanoscale topography on the near-surface microfluidic environment is available (Figure 2D-2).

Finally, the effect of various biological factors on hydrodynamics should also be considered in the context of bacterial attachment. Kirisits et al. (2007) reported that the critical amount of biomass required for full induction of QS of the population increased with flow rate in the environment. Topography-induced changes in microfluidic patterns may thus interfere with QS induction. Furthermore, bacterial attachment can be promoted by a catch-bond mechanism under low flow rate (Nilsson et al., 2006). However, this mechanism may fail under high flow rate, as high shear forces may break the hydrogen bonds between bacterial adhesins (e.g., FimH) and various surface components (e.g., mannose) (Thomas et al., 2002, 2008; Nilsson et al., 2006).

### Surface Wettability

The influence of the hydrophobicity of either bacterial cells or the surface on attachment and biofouling has been addressed in several studies. In general, hydrophobic interactions are favored between relatively hydrophobic bacteria and apolar surfaces, while hydrophilic cells prefer to adhere to hydrophilic surfaces (Hori and Matsumoto, 2010). The effect of hydrophobicity/ hydrophilicity is incorporated in physicochemical models, for example in the acid-base term (*F*^AB^) in the XDLVO theory (Feng et al., 2014, 2015). Surface hydrophobicity/hydrophilicity also affects surface wetting, which is a very important factor in bacterial attachment, but not considered by most physicochemical models.

#### Implication of Surface Topography-Chemistry Coupling

The wettability of a surface is determined by both the chemistry of the material and its physical topography (Genzer and Efimenko, 2006). Surface wettability is generally expressed by the contact angle of a liquid (θ), most often as the water contact angle (WCA). A given surface’s contact angle is related to the interfacial energies between various phases, including solid-liquid (*γ*^SL^), solid-vapor (*γ*^SV^), and liquid-vapor (*γ*^LV^), as described by Young’s equation:

(2) $$\cos(\theta )=\frac{\gamma^{\mathrm{SV}}-\gamma^{\mathrm{SL}}}{\gamma^{\mathrm{LV}}}$$

However, this equation is only valid for flat surfaces. For textured surfaces, the concept of intrinsic contact angle (θ_0_), which is the contact angle measured on an atomically smooth surface that is chemically identical to the textured surface of interest, becomes especially important. In case of textured surfaces, the apparent contact angles measured on a macroscopic scale rarely reflect the properties of the micro- or nanometric surface elements with which bacterial cells or their appendages actually interact. In the presence of surface topography, the measured contact angle can be different from what would be measured on a flat surface of the identical chemistry (Wenzel, 1936).

On a rough surface, the space between small protrusions may be filled with air instead of liquid, a state called the Cassie wetting regime. When the liquid completely wets the entire surface area, the Wenzel wetting regime is reached. The wetting state of a surface can change depending on surface topography. For example, the ratio of pore diameter to the inter-pore spacing, and the ratio of pore depth to the pore diameter appear to determine the wetting regime of nanoporous alumina surfaces (Ran et al., 2008; Feng et al., 2015). Hence, contact angles measured on micro- or nanostructured surfaces may not lead to accurate calculations of the actual surface energy, a quantity used in physico-chemical predictive models of bacterial attachment.

Decoupling surface energy and topographical contributions to bacterial attachment is very challenging. In a recent attempt to deconvolute the effects of surface energy and nanoscale topography on attachment, Zhang et al. (2018) exposed –OH and –CH_3_ terminated Si-based substrates, with or without nanoscale roughness, to *P. aeruginosa*. While the introduction of nanoscale topography (random spherical protrusions, *R*∼100 nm) enhanced biomass accumulation on substrates with either terminal group, the structure of the adhered biomass on nanorough substrates were clearly dependent on surface chemistry. On the contrary, Pegalajar-Jurado et al. reported no statistically significant difference between *E. coli* biomass on a smooth control and nanostructured surfaces (colloidal crystals, radius ∼200 nm, height ∼30 nm), for both hydrophobic (WCA = 90°) and hydrophilic (WCA = 37°) surfaces (Pegalajar-Jurado et al., 2015).

#### Effect of Surface Super-Hydrophobicity and Air Entrapment

The incomplete wetting of material surface, termed the Cassie-Baxter wetting state, allows air pockets entrapped in-between surface structures to create air-liquid interfaces that block bacteria from accessing the material surfaces, by limiting the material surface available for bacterial attachment. Scardino et al. (2009) compared underwater wetting behavior of superhydrophobic coatings with either nanoscale roughness, or with both nano- and micro-scale roughness. The authors used small angle X-ray-scattering, a technique sensitive to local changes in electron density contrast that results from partial or complete wetting of a rough interface. They found that the surface with just nanoscale roughness was able to deter the settlement of all of the tested microorganisms, whereas the surfaces with both nano- and micro-scale roughness did not show broad-spectrum fouling resistance. The authors attributed this difference in fouling to a noticeably larger amount of air entrapment at the nanostructure-only interface compared to the surfaces also with micro-scale roughness. More recently, Nguyen et al. (2018) found that microscale wrinkles were more effective in reducing attachment by *P. aeruginosa* and *S. aureus* than their nanoscale counterparts, due to the more substantial air-water interface of the microstructures, which effectively reduced the amount of solid-liquid interface accessible by the bacteria. Similarly, Yuan et al. (2017) attributed the reduced *E. coli* attachment to a fluorinated web topography with micrometric fibers and spacing (WCA = 168°, θ_0_ = 115°) to its substantial air-entrapment. In that study, only 3% of the fibrous surface area was interfaced with liquid and accessible by the bacteria, whereas the rest was occupied by the air-liquid interface.

It appears that bacterial cells approaching an air-liquid interface have no accessible sites for establishing a stable anchor via cellular appendages or other mechanisms, as it would be possible on a solid surface (Gibiansky et al., 2010; Epstein et al., 2012). Furthermore, the high surface tension of water (72 mN/m at 25°C) makes it extremely difficult for bacteria to penetrate the air-liquid interface. Therefore, high ratios of air-liquid to solid-liquid interfaces are favorable for low-fouling, due to the air entrapment. Surface topographies capable of stabilizing air-liquid interfaces with areas larger than the typical bacterium size are expected to be more effective in preventing fouling than those of much smaller feature sizes, as illustrated in Figure 2E. This happens because, compared to a nanotextured surface with intermittent air-liquid interfaces (Figure 2E-1), the continuous air-liquid interfaces present between the microscale features further minimizes the sites of accessible footholds for the bacterial cells (Figure 2E-3), thus interfering with bacterial attachment.

Inspired by experimental observations, attempts have been made to theoretically derive the design principles for stable air entrapment. Marmur (Marmur, 2006) proved theoretically that underwater air entrapment between surface structures is feasible and thermodynamically stable when the roughness ratio (*r*_f_) of surface topography is sufficiently high, where *r*_f_ is defined as the ratio between the true surface area of the solid and its projected area. Kaufman et al. (2017) developed a wetting model that allows to predict the wetting state under a liquid droplet on any type of textured surface, based on the intrinsic contact angle and the defined topography of the surface. They predicted that air entrapment can become thermodynamically stable when θ_0_ > 90° and *α*_max_ + θ_0_ > 180°, where *α*_max_ is the maximum slope of the surface topography. On the other hand, when θ_0_ < 90°, the liquid will eventually fully wet the surface, regardless of its topography. Therefore, very large intrinsic contact angles and re-entrant type topography are conducive of air entrapment. This principle can be used to engineer topographies that prevent bacterial attachment based on the air entrapment mechanism.

If air-liquid interfaces can effectively deter bacteria attachment, a reasonable question that arises is if this can also be achieved with liquid-liquid interfaces. Inspired by the *Nepenthes* pitcher plants, a group from Harvard University explored this possibility by infiltrating a surface nano-topography with a lubricant immiscible with the liquid medium, a novel construct that was called SLIPS (Slippery Liquid-Infused Porous Surfaces) (Wong et al., 2011; Epstein et al., 2012; Maccallum et al., 2015). SLIPS was found to have an approximately 35 times lower biomass accumulation of *P. aeruginosa* over a 7-day challenge, which was comparable with that of the best case scenario PEGylated substratum over a much shorter time frame (Epstein et al., 2012). Similar to the antifouling mechanism by air entrapment, bacteria near SLIPS lubricant-medium interface were not able to find a stable anchor, nor could they penetrate the interface due to the high liquid-liquid interfacial tension (Epstein et al., 2012). SLIPS was considered to possess several advantages over the air-entrapment: (1) complete coverage of solid substratum (Sotiri et al., 2016); (2) stability over a wide range of pressure, temperature, surface tension, and other conditions (Wong et al., 2011); (3) flexibility in the choice of lubricant liquid and substratum material (Wong et al., 2011).

### Topography-Induced Cell Ordering, Segregation, and Removal

It has been well-established that the interaction of individual bacterial cells with material surfaces are greatly influenced by surface topography (Hsu et al., 2013). Yet, how topography impacts at the individual cell level affect multicellular clusters and biofilms is just starting to be understood. In this section, we will first discuss the effect of static surface topographies and their dimensional scale on individual cell attachment and the organization of a multicellular community. After that, we will review recent developments in dynamic topographies and their potential for biofilm removal.

(1) *Static topography*. Surface topography of a scale comparable with microbial cell dimensions (about 1–2 μm) was recognized as a crucial and positive contributing factor to bacteria-surface interaction and bacterial attachment (Medilanski et al., 2002; Hochbaum and Aizenberg, 2010; Epstein et al., 2011; Friedlander et al., 2013; Hsu et al., 2013). In the study by Hsu et al. (2013) the orientation and subsequent attachment of microorganisms on silica with defined topography appeared to occur in a manner that maximized contact area between the cells and the surface. In a study on stainless steel surfaces, bacteria appeared to align parallel to scratches, and the width of these scratches seemed to play a role in their effective attachment: bacteria preferentially bound to 0.7 μm trenches, a size similar to the width of rod-shaped bacteria (Medilanski et al., 2002). A clear pattern of bacterial attachment was observed when the spatial distance of an array of surface posts approached the size of some Gram-negative and Gram-positive microorganisms: *P. aeruginosa*, *E. coli*, and *B. subtilis* (Hochbaum and Aizenberg, 2010). The features on these surfaces seemed able to direct cell patterning independent of the expression of appendages by the cells (Hochbaum and Aizenberg, 2010). The interstitial space between surface features (Epstein et al., 2011) and the depth of the features (Lai, 2018) were both found to be critical for bacterial cell patterning. For example, aggregation of *P. aeruginosa* cells on Si nanogratings was reduced to 20% of that on flat controls, because the cells were entrapped to the bottom of the Si nanogratings deeper than 500 nm, and consequently became unavailable for forming cell clusters (Lai, 2018). The patterning and ordering of early colonizers can interfere with natural biofilm development and organization (Hochbaum and Aizenberg, 2010), as some bacteria rely on contact-dependent signaling cascades to achieve cooperative communal functions (Blango and Mulvey, 2009). Moreover, bacterial mobility near surfaces can be influenced by the scale of the surface topographies. By carefully controlling surface chemistry, a group from Virginia Tech systematically probed the size effects of crystalline hemispherical topographies of 0.45–8 μm microsphere diameter on *P. aeruginosa* surface mobility and biofilm formation (Kargar et al., 2016; Chang et al., 2018). These studies revealed that the hemisphere diameter had a substantial influence on the net displacement of cells, the path of surface exploration, and consequently the spatial organization of biofilms.

By contrast, when the dimensions of surface topography are much larger than those of the microbial cells, the attachment behavior seems to be independent of the size of surface features. This was confirmed by exposing *P. aeruginosa* and *P. fluorescens* to silicon surfaces with rectangular grooves of 10–40 μm in width and 10 μm in depth, under flow conditions (Scheuerman et al., 1998). The attachment rate and surface coverage were not influenced by groove width, because from the vantage point of the bacteria the local topography they interact with is mostly flat. However, there was a significant difference in attachment behavior between motile and nonmotile strains, as the nonmotile strain could not effectively reach and attach to the bottom of the grooves, which demonstrates again that appendages have a crucial role in attachment, even under flow conditions.

Different than ordering of individual cells, segregation of bacterial colonies usually occurs on a larger length scale, and at a later stage, during biofilm development. A group from the University of Florida engineered a series of surfaces with microtopography inspired by the microbial resistant shark skin, coined Sharklet AF^TM^, consisting of parallel ribs (with 2 μm feature width and spacing, 3 μm height, and lengths ranging from 4 to 16 μm) arranged in a pattern of connecting rhombi (Carman et al., 2006). Poly(dimethyl siloxane) elastomer surfaces with Sharklet AF^TM^ patterns were found to disrupt the formation of *S. aureus* biofilms over 21 days without the use of bactericidal agents, possibly by obstructing the expansion of cell clusters and interfering with quorum sensing (Chung et al., 2007).

In summary, topography-induced cell ordering and colony segregation require surface topography scale comparable to or larger than a single cell. It should be noted that rod shaped bacteria can orient themselves either perpendicularly or parallel to a surface to meet the dimensional constraints and thermodynamic requirements (Figure 2F-3). Nanoscale features (<100 nm) are not large enough to accommodate the cells, and thus they do not facilitate the physical segregation of bacterial cells (Figure 2F-2).

(2) *Dynamic topography.* Although several topographical designs that can effectively reduce unwanted biofouling have been successfully developed, sustaining their effectiveness over extended periods of time is extremely challenging. This is mainly because over time various environmental (e.g., soil, or accumulation of a conditioning film) and biological (e.g., EPS production, cell-cell interactions) factors will slowly, but surely, mask the effective topographies (as discussed previously in the section on conditioning film) and eventually fail the antifouling design. This limitation can potentially be overcome by using dynamic topographies.

Dynamic topography as a biofilm prevention strategy has long existed in nature to prevent bacterial biofilm formation on wrinkled surfaces such as those of arteries, lung surfactant, or ureter, which are under constant challenge by biofouling (Pocivavsek et al., 2018). Inspired by nature, the concept of dynamic topography has been employed recently to prolong the effectiveness of antifouling topographical designs (Epstein et al., 2013; Shivapooja et al., 2013; Gu et al., 2016). Epstein et al. (2013) investigated bacterial attachment to dynamic substrates with regard to an array of parameters, including bacterial species and cell geometry, surface wrinkle length scale, amplitude of mechanical strain, intermittent vs. continuous strain cycles, and time. In the submicron topography range, the ∼1 μm, and ∼2 μm wide wrinkled valleys, close to the bacterial cell dimension, resulted in the largest attachment decrease for *P. aeruginosa* (∼80%) under continuous cyclic strain. Nonetheless, surface topography did not provide benefits compared to a flat static substrate over a challenge time of 72 h. The authors also noticed divergent effects of the dynamic topography on *P. aeruginosa, S. aureus, and E. coli* attachment and biofilm formation, possibly due to difference in cell geometry (i.e., rods vs. cocci) and types of flagellation (i.e., polar vs. peritrichous).

In addition to mechanical actuation, other external stimuli – including electrical voltage and air pressure – have also been explored as means to mitigate long-term biofouling (Shivapooja et al., 2013). Temperature change is an easy to use trigger for actuation, as it occurs naturally in many processes. Using biocompatible shape memory polymers (SMP) with hexagonal honeycomb-like surface patterns of repetition wavelength of about 80 μm, Gu et al. (2016) reported a remarkable 3-log reduction of established *P. aeruginosa* biomass in response to temperature cues, despite some limitations of the specific SMP used, as well as the storage and activation temperatures. Sidorenko et al. designed a dynamic actuation system based on the swelling and contraction of hydrogels driven by changes in humidity, which could be controlled to form a variety of reversibly actuated micropatterns with potential for antifouling applications (Sidorenko et al., 2007; Kirschner and Brennan, 2012). Environmentally-actuated, hybrid-material surfaces consisting of temperature-, humidity-, or pH-responsive hydrogels integrated with arrays of nano- or microstructures have been reviewed by Zarzar and Aizenberg (2014).

For some applications, such as surfaces of implant materials, the need to power the actuation mechanisms can pose significant challenges. Pocivavsek et al. (2019) designed and demonstrated the effectiveness of a self-cleaning and anti-thrombotic surface for vascular grafts that exploits the repeated wrinkling and unwrinkling driven by the pulsatile flow of the cardiovascular system, which could enable the continuous actuation of the grafts after they are implanted in the human body. Among the four tested lumen surface topographies – three with wrinkled lumen surfaces of various wavelengths (λ = 1000, 250, and 80 μm) and a smooth control – the actuated graft with the smallest wavelength (i.e., 80 μm) achieved the lowest thrombus formation, which was explained by the greater curvature of the small wavelength wrinkles (Pocivavsek et al., 2019). While this design has so far only been tested against platelet deposition, the physical mechanism underpinning the removal of biofilms composed of platelets – topography-driven delamination – may also be applicable to the removal of bacterial biofilms (Pocivavsek et al., 2018, 2019). Generally speaking, such mechanical approaches for *post hoc* biofilm dispersion open the door to extending or renewing the effectiveness of antifouling topographical designs and enhance their ability to combat fouling. Additionally, valuable insights may stem from the mechanistic investigation of bacterial responses to dynamic topographies, for instance information regarding regulation of genes that dictate the motile-to-sessile lifestyle switch.

### External Surface Stiffness

Although the impact of stiffness on bacterial attachment is still under-explored (Song et al., 2015), increasing evidence suggests this material property has a vital role in surface sensing and attachment. Lichter et al. found that adhesion of *S. epidermidis* to weak polyelectrolyte multilayer (PEM) thin films increased with the stiffness of these surfaces (with stiffness, expressed by Young’s modulus, ranging from 0.8 to 80 MPa), independent of the charge density, interaction energy, polymer surface roughness, and solution ion concentration (Lichter et al., 2008). Increasing evidence seems to suggest that the effect of stiffness on attachment strongly depends on the hydrophobicity/hydrophilicity of the substrate surface.

(1) *Stiffness of hydrophobic surfaces*. The limited number of studies available in literature on this topic seem to suggest a decreasing trend in attachment with increasing surface stiffness. Song et al. prepared a series of hydrophobic poly(dimethylsiloxane) (PDMS) surfaces with stiffness ranging from 0.1 to 2.6 MPa, and exposed them to *E. coli* and *P. aeruginosa* cells (Song and Ren, 2014). They reported that the initial attachment of both species was higher on the softer PDMS. A follow-up study from the same group revealed that cells attached to soft PDMS surfaces were less motile than those attached to the stiff surfaces, which was attributed to the expression of *motB*, a gene encoding flagellar motors (Song et al., 2017). The same group later demonstrated that the level of intracellular cyclic dimeric guanosine monophosphate (c-di-GMP), which plays a key role in regulating the transition from planktonic growth to biofilm formation, was higher when wild type *P. aeruginosa* cells attached to soft PDMS surfaces compared to stiff surfaces (Hengge, 2009). This suggests that *P. aeruginosa* cells may be able to actively sense material stiffness via *OprF* during attachment, and respond by changing c-di-GMP level, whereas mutation of the *oprF* gene abolished *P. aeruginosa* mechanosensing to surface stiffness during attachment (Song et al., 2018). Attachment of cells to stiff PDMS has also been associated with upregulated stress response, including augmented resistance to several classes of antibiotics (Song and Ren, 2014). Future research is needed to probe the bacterial responses to the stiffness of a wider range of hydrophobic surfaces, and deepen our understanding of this phenomenon.

(2) *Stiffness of hydrophilic surfaces.* High stiffness has been shown to increase bacterial attachment and biofilm formation by several studies (Lichter et al., 2008; Kolewe et al., 2015; Kolewe et al., 2018), but the opposite trend was also reported (Wang et al., 2016). Polyacrylamide (PAAm) and poly(ethylene glycol) dimethacrylate (PEGDMA), two hydrophilic surfaces of different stiffness, were challenged with *S. aureus* in two separate studies, and *S. aureus* biomass negatively correlated with stiffness for PAAm, but positively for PEGDMA (Kolewe et al., 2015; Wang et al., 2016). It should be noted that the stiffness range probed in the PAAm study (i.e., 10^−2^–10^−1^ kPa) was orders of magnitude lower than that in the PEGDMA study (i.e., 10^2^–10^3^ kPa). This difference in the magnitude of substrate stiffness, in addition to differences in terms of polymer chemistry, flow conditions, methods for stiffness determination, and liquid medium, may be responsible for the apparent conflicting trends of bacterial attachment on hydrophilic polymers. Future research is needed to cover a broad stiffness range, and also to elucidate the bacterial molecular mechanisms triggered in response to hydrophilic surface of a range of stiffness (e.g., changes in c-di-GMP level) before final conclusions can be drawn on the stiffness-attachment relationship for hydrophilic surfaces.

Another important, yet little understood, aspect of the stiffness-attachment relationship is the relative contribution of external surface vs. subsurface stiffness. By masking the bulk stiff PEM substrate (Young’s modulus, *E* = 80 MPa) with a *single* bilayer of compliant PEM (*E* = 0.8 MPa) and vice versa, Lichter et al. reported a near reversal of effective surface stiffness (measured by AFM nanoindentation to maximum depths of less than 20 nm), which consequently reversed the trend of *S. epidermidis* attachment (Lichter et al., 2008). These results suggest that bacterial mechanosensing may be significantly affected by the first tens of nanometers of depth of a substrate surface, whereas the influence from the deeper layers of the substrate is comparatively rather limited. In a more recent study, Kolewe et al. (2018) prepared hydrophilic poly(ethylene glycol) (PEG) surfaces with three levels of Young’s moduli: soft (20 kPa), intermediate (300 kPa), and stiff (1000 kPa), each at three thickness levels: thin (15 μm), medium (40 μm), and thick (150 μm) (Kolewe et al., 2018). For all thickness levels, attachment by *S. aureus* and *E. coli* decreased with decreasing PEG stiffness. The effect of stiffness on reducing attachment became substantially greater with increasing PEG thickness, even though no significant difference in local Young’s modulus was identified by the AFM nanoindentation. When considering the conclusions of these two studies, an intriguing question arises: if bacterial mechanosensing is effective only within a few tens of nanometers of the substrate, as shown by Lichter et al., how could the bacteria in the study by Kolewe et al. ‘respond’ so drastically to changes in thickness that was three orders of magnitude deeper than the stiffness-sensing range? Clearly, more research is needed to answer this question.

It should also be noted that so far the available studies on stiffness invariably used polymeric substrates, which inherently comprise mesh-like surface topographies that feature nano- to subnanoscale pores (Kolewe et al., 2015; Wang et al., 2016; Canal et al., 1989). For example, stiffer polymer surfaces usually possess a higher network density than their softer counterparts, which consequently lead to a higher density of functional groups, with which liquid medium and bacterial cells can interact. Since the nanoporous topography is an implicit variable that covaries with stiffness, it may contribute to the observed bacterial attachment trend along with stiffness. Overall, the experimental evidence available at the time this paper was written suggests that material properties including hydrophobicity, the range of stiffness, and the characteristics of subsurface materials, can all influence the trend of bacterial attachment regarding external surface stiffness. Advances in nano-characterization techniques, such as high resolution atomic force microscopy and small-angle X-ray or neutron scattering (Mochizuki et al., 2014), may open up new avenues for decoupling the effect of topography from surface stiffness, and help explain some of the inconsistencies observed in the stiffness studies.

## Overall Perspective and Conclusions

Bacterial attachment is a complex process determined by the interplay between surface properties, biological factors, and environmental conditions. In this paper, we reviewed some of the most recent advances in understanding bacteria-surface interactions, with a focus on how surface topography and its scale affect this process, through both biological and nonbiological mechanisms.

The bacterial attachment to a surface is a complex multistage process that involves locating, approaching, and sensing the proximity of the surface. Within each stage, bacterial cells constantly take in physicochemical or biological signals from their immediate surroundings and respond accordingly. Since surface topography can shape the near-surface microenvironment, it inevitably plays a central role in defining the outcome of bacterial attachment. Scientific efforts to elucidate the effect of topography on bacterial attachment often led to contradictory conclusions, and some of the main reasons could be: (i) important topographical information is lost when roughness is used as the sole descriptor of surface topography; (ii) topographical effects are compounded with the effects of other physicochemical factors (e.g., surface chemistry); (iii) different antifouling mechanisms may “switch on” at different topographical scales. The first two reasons can be addressed by a precise description of surface topography and by carefully controlling the surface properties that may interfere with attachment (Figure 1). The third reason represents more of a challenge. In general, micrometric scale topography, comparable to that of bacterial cells, impacts attachment via hydrodynamics, topography-induced cell ordering, and air-entrapment, whereas nanometric topography impacts attachment via alteration of chemical gradients, physicochemical force fields, and cell membrane deformation (Figure 2). Multiple anti-attachment mechanisms are likely involved at the same time; while some of these mechanisms are turned “on” at a certain scale, others may be turned “off.” To complicate matters more, these mechanisms have different ranges of action – e.g., the effect of hydrodynamics is more far-reaching than that of the XDLVO forces. The presence of a conditioning film often leads to changes in surface chemistry and surface topography, and therefore the lifetime of nanoscale surface topography after exposure to complex media must be considered, as nanoscale topography is susceptible to masking by particles or even molecules present in the environment. Among all the mechanisms discussed, the effect of material stiffness on attachment is the least understood and requires further investigation. Another area that can benefit from further investigation is the bacterial response to the above-mentioned factors via regulation of gene expression. As several studies have already demonstrated, advances in understanding and controlling bacteria-surface interactions via surface topography can provide exciting solutions to biofilm control in many areas that directly affect human health and life including food processing, water treatment, medicine, or marine applications, to name a few.

## Author Contributions

All authors contributed to the development of the manuscript, with YC and CM assuming equal roles in the final editing and submission.

## Conflict of Interest Statement

The authors declare that the research was conducted in the absence of any commercial or financial relationships that could be construed as a potential conflict of interest.
